# Evaluating mismatch repair deficiency in colorectal cancer biopsy specimens

**DOI:** 10.1007/s00418-023-02202-8

**Published:** 2023-06-07

**Authors:** F. Grillo, M. Paudice, A. Gambella, S. Bozzano, S. Sciallero, A. Puccini, S. Lastraioli, M. Dono, P. Parente, A. Vanoli, V. Angerilli, M. Fassan, L. Mastracci

**Affiliations:** 1 IRCCS Ospedale Policlinico San Martino, Largo Benzi 10, 16132 Genoa, Italy; 2 Pathology Unit, Department of Surgical Sciences and Integrated Diagnostics (DISC), University of Genoa, Genoa, Italy; 3 Department of Medical Sciences, University of Turin, Turin, Italy; 4 Medical Oncology Unit 1, IRCCS Ospedale Policlinico San Martino, 16132 Genoa, Italy; 5 Medical Oncology and Hematology Unit, IRCCS Humanitas Research Hospital, Humanitas Cancer Center, Rozzano, Milan, Italy; 6 Molecular Diagnostic Unit, IRCCS Ospedale Policlinico San Martino, Genoa, Italy; 7 Pathology Unit, Fondazione IRCCS Casa Sollievo della Sofferenza, San Giovanni Rotondo, FG Italy; 8 Department of Molecular Medicine, Unit of Anatomic Pathology, University of Pavia, Pavia, Italy; 9 Anatomic Pathology Unit, Fondazione IRCCS San Matteo Hospital, Pavia, Italy; 10 Department of Medicine (DIMED), Surgical Pathology Unit, University Hospital of Padua, Padua, Italy; 11 Veneto Institute of Oncology IOV - IRCCS, Padua, Italy

**Keywords:** Colorectal cancer, Microsatellite instability, Mismatch repair, Lynch syndrome, Immunohistochemistry, Pitfalls

## Abstract

Mismatch repair (MMR) testing on all new cases of colorectal cancer (CRC) has customarily been preferably performed on surgical specimens, as more tissue is available; however, new clinical trials for the use of immune checkpoint inhibitors in the neoadjuvant setting require MMR testing on biopsy samples. This study aims at identifying advantages, disadvantages and any potential pitfalls in MMR evaluation on biopsy tissue and how to cope with them. The study is prospective-retrospective, recruiting 141 biopsies (86 proficient (p)MMR and 55 deficient (d)MMR) and 97 paired surgical specimens (48 pMMR; 49 dMMR). In biopsy specimens, a high number of indeterminate stains was observed, in particular for MLH1 (31 cases, 56.4%). The main reasons were a punctate nuclear expression of MLH1, relatively weak MLH1 nuclear expression compared to internal controls, or both (making MLH1 loss difficult to interpret), which was solved by reducing primary incubation times for MLH1. A mean of  ≥﻿ 5 biopsies had adequate immunostains, compared to ≤ 3 biopsies in inadequate cases. Conversely, surgical specimens rarely suffered from indeterminate reactions, while weaker staining intensity (*p* < 0.007) for MLH1 and PMS2 and increased patchiness grade (*p* < 0.0001) were seen. Central artefacts were almost exclusive to surgical specimens. MMR status classification was possible in 92/97 matched biopsy/resection specimen cases, and all of these were concordant (47 pMMR and 45 dMMR). Evaluation of MMR status on CRC biopsy samples is feasible, if pitfalls in interpretation are known, making laboratory-specific appropriate staining protocols fundamental for high-quality diagnoses.

## Introduction

Understanding the clinical and molecular heterogeneity of colorectal cancer (CRC) is the basis for an expanding array of patient-tailored treatment options. While the majority of CRCs follow the chromosomal instability pathway, approximately 15% show microsatellite instability (MSI) secondary to defective mismatch repair (MMR) mechanisms, thus leading to errors in replication and accumulation of mutations. Genes involved are *MLH1*, *MSH2*, *MSH6* and *PMS2* as well as *EPCAM* (found upstream of *MSH2*), and these may be mutated in the germline setting (such as in Lynch syndrome—LS) or silenced through *MLH1* promoter methylation, which is the basis of most sporadic MSI CRCs. LS is the most frequent heritable cancer syndrome (found in about 2–3% of CRC patients), and 20% of all MSI CRC are LS-associated (as well as other cancer types such as endometrium, gastric, small bowel, upper tract urothelial cancers, etc.).

In the last 10 years or so, international guidelines from various speciality societies (EGAPP working group 2009; Stjepanovic et al 2019; Weiss et al. 2021) have advocated MMR/MSI testing on all new cases of CRC in an initiative called Universal Screening (US) for LS detection. The basis for these recommendations is that, by identifying LS patients and their LS kindreds, new cancer diagnoses will be reduced though screening and prevention programmes. US by MMR, however, needs to include further steps to reduce the number of identified patients who are sent for germline testing, as most MSI CRCs will be, as mentioned above, sporadic in nature. About 60% of *MLH1* promoter-methylated sporadic cases will harbour *BRAF* V600E mutations, and consequently, *BRAF* mutation and/or *MLH1*-methylation status analysis can be used as a means of excluding patients from genetic testing (Tibiletti et al. 2022).

Besides LS identification, MMR/MSI testing has become increasingly important for clinical decision-making, as a prognostic and predictive indicator. MMR/MSI status has been shown to be associated with better survival for stage II and low-risk stage III CRCs (Popat et al. 2005; Sinicrope et al. 2021), while data remain controversial on the prognostic value of MMR/MSI status in patients with metastatic disease (MMR/MSI is found in only 5% of metastatic CRCs) (Venderbosch et al. 2014; Innocenti et al 2019). MMR/MSI testing also seems to be fundamental in the choice of adjuvant treatment in stage II and III CRCs. Indeed, adjuvant fluoropyrimidine monotherapy seems to show limited benefit in MMR-deficient (dMMR)/MSI CRC, and, if adjuvant treatment is decided upon (mostly for some high-risk stage II and stage III CRCs, though there is no agreement between American and European guidelines), this should be a combination of fluoropyrimidine and oxaliplatin. Lastly, and perhaps most importantly, MMR/MSI testing is the prerequisite biomarker for treatment with immune checkpoint inhibitors (ICIs) in CRC. While ICI efficacy has been demonstrated in metastatic dMMR/MSI CRCs (Overman et al. 2017; Diaz et al. 2022), leading to swift regulatory approval, its use in the neoadjuvant setting is the focus of the most recent clinical trials (Ludford et al. 2023). In the NICHE-2 trial (Chalabi et al. 2022), a major pathologic response (less than 10% of residual viable tumour cells) rate of 95%, including 67% pathologic complete responses, was achieved in a large cohort of dMMR CRC patients treated with short-term neoadjuvant nivolumab plus ipilimumab and subsequent surgery within 6 weeks. Furthermore, in a prospective phase II study (Cercek et al. 2022), 12 patients with dMMR rectal cancer were treated with an anti-PD-1 antibody, dostarlimab, and a watch-and-wait strategy (without chemotherapy or surgical intervention) was applied if they showed clinical complete response. Though some patients had short follow-up, all showed complete clinical response, and this has generated great interest, especially with regard to organ-sparing disease control.

The identification of dMMR/MSI CRC is, therefore, becoming increasingly widespread (though far from ubiquitous) and is possible using immunohistochemical (IHC) detection of MMR proteins, polymerase chain reaction (PCR) amplification of specific microsatellite repeats or validated next-generation sequencing (NGS) assay. These techniques are equally valid in detecting dMMR or MSI CRC, and various concordance studies show overlapping results, even though IHC MMR testing is the most economical and widely available method (Bartley et al. 2022; Wang et al. 2022; Luchini et al. 2019).

While MMR testing is most often carried out on surgical samples, studies have shown that biopsy tissue is adequate for MMR testing, and indeed, may even be superior considering that samples are fixed as soon as they are taken, showing minimal cold ischemia, and their small size means that hypo-fixation is generally not a problem (O’Brien et al. 2018; Kumarasinghe et al. 2010; Shia et al. 2011; Warrier et al. 2011; Vilkin et al. 2015). Furthermore, optimal concordance between MMR status on biopsy and surgical samples has been observed, making biopsy tissue reliable for MMR testing (Kumarasinghe et al. 2010; Shia et al. 2011). Are there drawbacks for MMR testing of biopsy tissue? One such drawback is that biopsy tissue may vary substantially in terms of size and cellularity and may be the only tissue obtained from the patient (e.g. in patients presenting with metastatic disease, who will not undergo surgical resection), making it extremely precious with regard to other molecular tests which may be or become important for therapeutic management (e.g. *KRAS*/*NRAS* testing) (Hale et al. 2015). Importantly, while the number of neoplastic biopsies is recommended for gastric and oesophageal cancers (Lordick et al. 2022) (5–8 samples, usually about 5–6; Gullo et al. 2015; Grillo et al. 2013), no such number is provided for colorectal cancer biopsy sampling (van de Velde et al. 2014).

Until now, having pre-surgical knowledge of CRC MMR status was important pre-eminently in those rare LS patients for whom surgical strategy could vary. Conversely, at the present time, the recent neoadjuvant immunotherapy treatment trials require MMR testing on biopsy samples, thus meaning that pathologists must change their MMR/MSI testing strategies. In our institution, US by IHC MMR testing was introduced in 2012 on all new CRC diagnoses on surgical resection samples, but we have recently shifted to MMR testing of all new CRC biopsy cases. The present study aims at identifying, on a prospective case series of biopsies for any CRC and a retrospective series of only dMMR CRC biopsies, the potential pitfalls in MMR evaluation on biopsy tissue and how to cope with them.

## Materials and methods

### Case selection

In January 2022, a new, prospective institutional protocol for the assessment of MMR by immunohistochemistry for MLH1, PMS2, MSH2 and MSH6 on biopsy tissue was put into place following multidisciplinary team discussion, and all findings were added to the pathology report in terms of MMR deficiency or proficiency (dMMR versus pMMR—as well as which protein/protein couple lost expression). A case series comprising 103 unselected, prospective, endoscopic biopsy cases diagnostic for CRC between January 2022 and September 2022 were identified from the pathology database of the University of Genova/IRCCS Ospedale Policlinico San Martino, Genova, Italy. Fifty-nine of the 103 patients had subsequent surgical resections in the same institution, and these cases were also selected.

**Fig. 1:**
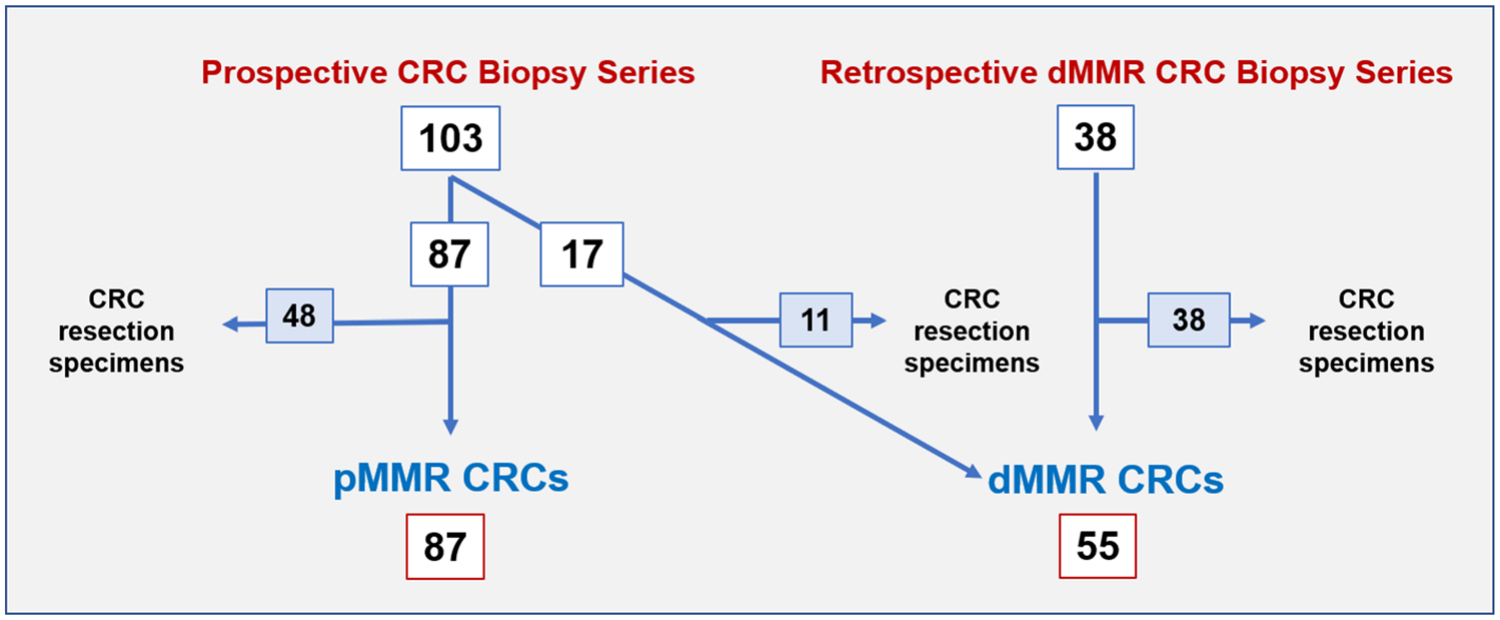
Schematic showing study design. Numbers in boxes: number of MMR tested biopsy cases in white, and number of biopsy cases which also have a matched resection specimen in blue. *CRC* colorectal cancer; *dMMR* mismatch repair-deficient; *pMMR* mismatch repair-proficient

In order to expand the number of dMMR biopsy cases, a second case series, retrospective in nature, identified endoscopic biopsies from 38 dMMR CRC resection specimens from March 2020 to December 2021 from the same pathology database. Figure 1 shows the study design.

Clinical data, including patient age, gender, site of tumour and neoadjuvant (chemoradiation) treatment for rectal cancers, were obtained from the pathology database. All data were anonymized. The application of the LS algorithm is being performed within the framework of the Italian ItaLynch Study (an ongoing prospective, observational multicentric study). Ethics committee approval was obtained at the University of Genova/IRCCS Ospedale Policlinico San Martino, Genova, Italy, number 101/2021 (1 March 2021). The study was conducted in accordance with the ethical principles of the Declaration of Helsinki.

### Histologic analysis

All selected samples were initially evaluated on haematoxylin and eosin-stained sections; only cases with invasive adenocarcinoma were included. The grade and morphology of the CRC were identified both on biopsy tissue (where possible) and on the resection specimen when available. Evaluation was performed using a Leica DM 2000 LED optical microscope (Leica Microsystems Inc., Wetzlar, Germany). All microphotographs were captured using the Leica Microsystems Flexacam C1 (Full-HD 1920 × 1080 pixels; Leica Microsystems Inc., Wetzlar, Germany) and Leica Application Suite X (LAS X) acquisition software (Leica Microsystems Inc., Wetzlar, Germany). Images were captured with varying magnification—4×, 40× and 63×.

The number of available endoscopic biopsies, as well as how many of these contained invasive adenocarcinoma, were noted for each patient.

### Immunohistochemistry (IHC)

IHC was performed using the BenchMark Ultra (Ventana Medical Systems, Roche Diagnostics Division, Hoffmann-La Roche Ltd, Basel, Switzerland) automated immunostainer and visualization of the antibody-antigen reaction was via the indirect biotin-free method, ultraView Universal diaminobenzidine detection kit (Ventana Medical Systems, Roche Diagnostics Division, Hoffmann-La Roche Ltd, Basel, Switzerland). The slides were counterstained with haematoxylin. The following antibodies were used: hMLH1 (M1 clone, Ventana, 60 min heat pre-treatment, incubation time 80 min), hPMS2 (EPR3947 clone, Cell marque, 30 min heat pre-treatment, incubation time 40 min), hMSH2 (G219-1129 clone, Cell marque, 30 min heat pre-treatment, incubation time 60 min) and hMSH6 (clone 44, Ventana, 30 min heat pre-treatment, incubation time 20 min). Proof of validation for antibodies was present in the technical specification inserts provided by the manufacturers. Technical validation of adequacy of IHC was possible with external on-slide control composed of colonic mucosa (Bragoni et al. 2017) and/or internal control of colonic mucosa and/or tumour-associated stroma. The IHC laboratory at our institution has taken part in the UK NEQAS (United Kingdom National External Quality Assessment Service) quality assurance assessment runs (2022) for MMR testing.

### IHC staining evaluation and interpretation

Each case (both biopsy and surgical resection specimens) was evaluated blindly by expert gastrointestinal pathologists; any differences were resolved by consensus.

**Fig. 2:**
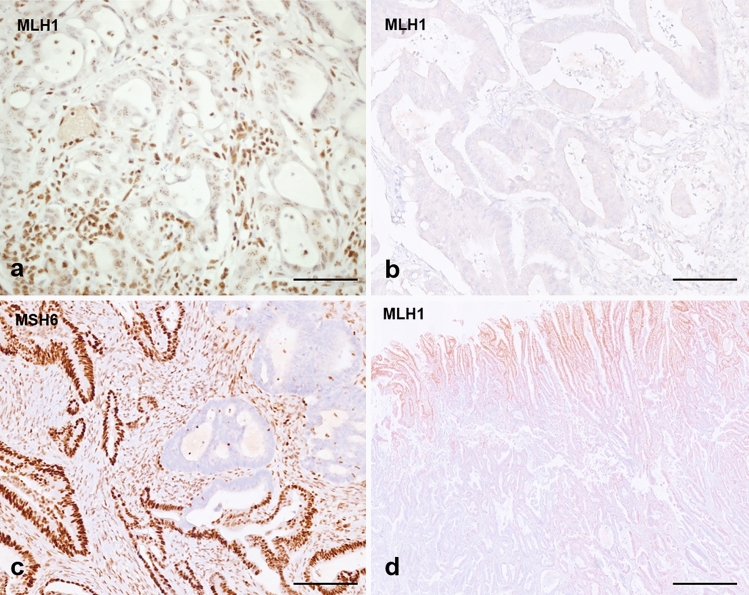
**a** MMR-deficient biopsy case showing complete loss of MLH1 expression in nuclei with minimal punctate nuclear expression—this type of expression is usually interpreted as loss of expression and leads to a diagnosis of deficient MMR. **b** Case of inadequate MMR immunostaining (surgical case), as no nuclear expression is seen in either neoplastic or internal control (stromal and inflammatory cells); this case was MSS by MSI testing. **c** Case of MMR-deficient tumour with loss of expression of MLH1 and PMS2 (not shown) and sub-clonal loss of MSH6 (surgical case). **d** pMMR colorectal cancer resection specimen showing central artefact with preserved expression in neoplastic and control cells towards the periphery, and loss of expression of both neoplastic and control cells in the central part of the tumour (probably due to hypo-fixation of the central area). Scale bar in **a**, **b** and **c**—50 µm, magnification ×40; scale bar in **d**—200 µm, magnification ×4

Initial evaluation at routine light microscopy permitted categorization (Fassan et al. 2020) of each case in: MMR-proficient (pMMR), MMR-deficient (dMMR), indeterminate and inadequate. pMMR was thus defined when tumour nuclear staining was comparable to the internal or external controls. dMMR was defined as complete loss of nuclear expression of tumour nuclei with retained expression in internal control nuclei. Staining was deemed indeterminate when tumour nuclei showed focal (< 10% of surface) or weak expression, fainter than control nuclei (Sarode and Robinson 2019). Further indeterminate cases were those showing punctate nuclear expression (Fig. 2a) or cytoplasmic expression with possible nuclear loss difficult to reliably assess. Indeterminate cases were sent to MSI testing with PCR unless other tissue (e.g. resection specimen) was available for repeat testing by IHC. Finally, staining was considered inadequate if no nuclear staining was observed in either the tumour nuclei or control nuclei (Fig. 2b) and immunostaining was repeated or sent to MSI testing. MMR status categorization on biopsies is shown in Table 1.

**Table 1:** Categorization scheme for MMR staining interpretation

MLH1	PMS2	MSH2	MSH6	Categorization
Preserved	Preserved	Preserved	Preserved	pMMR
Lost	Lost	Preserved	Preserved	dMMR
Preserved	Lost	Preserved	Preserved	
Preserved	Preserved	Lost	Lost	
Preserved	Preserved	Preserved	Lost	
Indeterminate or inadequate		Lost	Lost	Probably dMMR—repeat/confirm on MSI
Lost	Lost	Indeterminate or inadequate		
Inadequate/indeterminate		Inadequate/indeterminate		Unclassified—repeat/send to MSI testing

*pMMR* proficient MMR; *dMMR* deficient MMR; *MSI testing* microsatellite instability testing by PCR

All cases, both biopsy and surgical specimens, were evaluated for staining intensity, patchiness (patchy staining distribution, probably due to fixation artefacts, with reduced immunostaining in neoplastic cells and/or in internal controls]) and central artefact (reduction in central immunostaining).

*Staining intensity* was classified as “strong” when staining of non-tumour nuclei was clearly visible and intense. “Weak” was assigned to cases for which control nuclear expression was visible but faint. If staining of control nuclei was absent, this was deemed inadequate as stated above.

*Patchiness* of staining was assessed, and scores were assigned according to distribution of nuclear staining: score 0 when no staining patchiness was observed (staining in 100% of sample); score 1 when mild patchiness was observed (staining in 70–99% of sample), score 2 when moderate patchiness was seen (staining in 50–69% of sample) and finally score 3 patchiness when staining was seen in 10–49% of the sample. Cases showing < 10% of staining in the tumour were considered indeterminate and further action was taken (see above). Any true heterogeneity (described as confluent areas of staining loss involving multiple adjacent glands, with preserved internal control, accompanied by confluent areas of staining retention with stark contrast between areas) was noted (McCarthy et al. 2019; Loupakis et al. 2019) (see Fig. 2c).

Cases were then analysed for *central artefact*, which is defined as the presence of a rim of adequately stained tissue towards the outer surface but reduced/inadequate expression observed in the central part of the tissue (Grillo et al. 2021). This artefact is mostly due to inadequate fixation of tissue, leading to hypo-fixed areas in the central part where formalin has not permeated sufficiently, and was scored as follows: absent, mild (when most of the section was stained except for the innermost portion of tissue) and marked (when only the outermost rim was stained and most of the section was weakly or inadequately stained) (see Fig. 2d).

### Modification of MLH1 immunohistochemistry protocols on biopsy tissue

As many dMMR MLH1 stained biopsies showed relatively weak expression/punctate MLH1 staining (see results section), modified immunostaining protocols were tested to identify the best staining protocol for biopsy samples. Various combinations of reduced pre-treatment times and/or incubation times were tested, and compared with the standard used in our laboratory (M1 clone, Ventana, 60 min heat pre-treatment, incubation time 80 min; this staining protocol was tailored for the fixation and processing times of surgical specimens in our institution).

In particular:

- M1 clone, Ventana, 60 min heat pre-treatment, incubation time 40 min (protocol A);
- M1 clone, Ventana, 30 min heat pre-treatment, incubation time 80 min (protocol B);
- M1 clone, Ventana, 30 min heat pre-treatment, incubation time 40 min (protocol C).

Testing was performed on 24 biopsy specimens from the dMMR retrospective series with sufficient tissue available and which had undergone surgical resection (so that there was no risk of loss of tissue).

### MSI analysis in problematic cases, *BRAF* mutation evaluation and *MLH1* promoter methylation status in CRC with loss of MLH1/PMS2

MSI analysis was performed on cases with indeterminate/inadequate IHC; as per the LS screening protocol, cases with loss of MLH1/PMS2 underwent reflex testing for *BRAF* mutation analysis, and subsequently, if *BRAF* was found to be wild-type, *MLH1* promoter methylation status was assessed.

Genomic DNA was extracted from formalin-fixed paraffin-embedded (FFPE) tumour tissue sections using the QIAamp DNA FFPE tissue kit (Qiagen, Hilden, Germany) and used in the following molecular tests:

1. MSI analysis was performed by amplification of five mononucleotide microsatellites markers (Bethesda revised panel) and analysis of fluorescent fragments on an AB 3500 Genetic Analyzer (Thermo Fisher Scientific, Inc., Waltham, MA, USA). MSI was defined as indicated (Umar et al. 2004).
2. *BRAF* Val600Glu determination was performed by real-time PCR amplification (Easy PGX Thyroid Real Time PCR kit—1% sensitivity [Diatech Pharmacogenetics srl, Jesi, Ancona, Italy]).
3. *MLH1* promoter methylation by amplification of five CpG sites within the *MLH1* gene promoter region (positions –248 and –178, Deng C-region upstream NM_000249.3, 246 base pairs before ATG) in real-time PCR of sodium bisulfite-treated genomic DNA. Analysis of methylation CpG sites was performed by pyrosequencing on PyroMark Q96 ID and PyroMark CpG software (Qiagen, Hilden, Germany). Cases with ≥ 16% of methylation CpG islands were considered methylated (Adar et al. 2017).

### Statistical analysis

Descriptive statistics was applied to demographic and histologic characteristics, including median and range of number of biopsies per patient/median and range of biopsies with invasive cancer. Differences in staining intensity and patchiness between immunostains and between endoscopic biopsies and surgical specimens for each stain were calculated using the chi-squared test. A cut-off of *p* < 0.05 indicated a significant difference between groups.

## Results

Pooling both prospective and retrospective biopsy series, a total of 141 biopsies were evaluated from 140 patients (one patient had two sets of biopsies taken from two synchronous cancers). From this pooled case series, 65 (46.4%) were men while 75 (53.6%) were women; median patient age was 77 years (range 33–91). Median number of biopsy samples was 6 (range 1–13) while median number of biopsy samples with diagnostic adenocarcinoma was 5 (range 1–13).

Considering the pooled biopsy series, 86 cases were identified as pMMR while 55 cases were identified as being dMMR.

### pMMR biopsies from the prospective series

The 86 biopsy cases (see Table 2) were diagnostic in all but two indeterminate (one for MLH1 and one for PMS2) and one inadequate for PMS2 (all MSS at PCR analysis). With regard to staining *intensity*, PMS2 showed the highest number of weak stains, and this was statistically significant both comparing all stains together (*p* < 0.0001) and solely comparing PMS2 and MLH1 (*p* = 0.0002). Score 1 *patchiness* was seen solely for MLH1 and PMS2, while score 2 and 3 cases were seen only for PMS2. These differences were statistically significant when comparison was made between all antibodies (*p* < 0.0001), while no differences were seen between MLH1 and PMS2 (*p* = 0.155). MSH2 and MSH6 posed no problems whatsoever with regard to staining or patchiness. All biopsy samples were free from central, reduced staining artefacts.

**Table 2:** Comprehensive table showing, for both biopsy series and their matched resection specimens, categorization, quality, patchiness and central artefact for MMR immunoreactions with MLH1, PMS2, MSH2 and MSH6 antibodies

		Category			Staining intensity		Patchiness				Central artefact		
	Lost	Preserved	Indeterminate	Inadequate	Strong	Weak	0	1	2	3	0	1	2
pMMR BIOPSY SERIES 86 (prospective)													
MLH1	0	85	1	0	85	1	69	17	0	0	86	0	0
PMS2	0	84	1	1^a^	70	15	66	14	4	1	86	0	0
MSH2	0	86	0	0	86	0	86	0	0	0	86	0	0
MSH6	0	86	0	0	86	0	86	0	0	0	86	0	0
dMMR BIOPSY SERIES - 55 (17 prospective and 38 retrospective)													
MLH1	20	3	31	1^a^	44	10	53	1	1	0	55	0	0
PMS2	46	2	1	6^a^	38	11	55	0	0	0	55	0	0
MSH2	1	54	0	0	50	5	55	0	0	0	55	0	0
MSH6	3	52	0	0	50	5	55^b^	0	0	0	55	0	0
pMMR SURGICAL RESECTION SERIES - 48 (prospective)													
MLH1	0	48	0	0	44	4	13	17	12	6	32	4	12
PMS2	0	48	0	0	39	9	13	11	14	10	36	2	10
MSH2	0	48	0	0	47	1	38	5	2	3	45	0	3
MSH6	0	48	0	0	47	1	35	8	2	3	43	2	3
dMMR SURGICAL RESECTION SERIES - 49 (11 prospective and 38 retrospective)													
MLH1	44	3	1	1^a^	33	15	25	9	8	6	38	1	9
PMS2	45	3	0	1^a^	38	10	30	4	9	5	40	3	5
MSH2	1	48^c^	0	0	44	5	25	7	8	9	39	3	7
MSH6	2	47^d^	0	0	48	1	29	5	7	8	42	2	5

*pMMR* proficient MMR; *dMMR* deficient

^a^Inadequate cases were not evaluated for other variables

^b^One biopsy case showed loss of MLH1/PMS2 with sub-clonal loss of MSH6 on biopsy (confirmed on the resection specimen)

^c^ Two cases showed sub-clonal loss

^d^ Three cases showed sub-clonal loss

### dMMR biopsies from the prospective and retrospective series

**Fig. 3:**
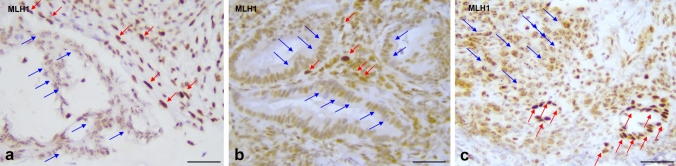
Biopsy cases of colorectal cancers with MMR deficiency but indeterminate MLH1 expression (MSI-H by PCR analysis and loss of MLH1 on subsequent resection specimens). **a** MLH1/PMS2 MMR-deficient colorectal cancer showing reduced nuclear MLH1 immunostaining with punctate nuclear expression (blue arrows) and preserved, intense, nuclear control in stromal cells (red arrows); **b** MLH1/PMS2 MMR-deficient colorectal cancer showing relatively weak nuclear expression of MLH1 (blue arrows) which is only slightly reduced compared to preserved, intense, nuclear control in stromal cells (red arrows); **c** MLH1/PMS2 MMR-deficient colorectal cancer showing relatively weak nuclear expression and nuclear punctate expression of MLH1 (see blue arrows); red arrows show internal control non-neoplastic crypts with preserved intense nuclear expression. Scale bar—50 µm, magnification ×40

A total of 55 biopsy cases were dMMR (see Table 2): 17 from the prospective series (17/103—15.5%) and 38 from the retrospective series. MLH1 was preserved in three cases and clear loss of expression was seen in 20 cases, while 32 cases showed either indeterminate (31 cases) or inadequate (one case) expression. Prospective indeterminate and inadequate cases (which all showed clear nuclear expression of the MSH2/MSH6 pair) were sent to MSI testing by PCR and these were all diagnosed as MSI-H. The main reasons for such a high number of indeterminate biopsies were a punctate (dot-like) nuclear expression of MLH1 of variable intensity (seven cases), a reduced (but clearly visible) nuclear expression compared to internal controls (but no complete loss—seven cases) or both (17 cases)—see Fig. 3.

PMS2 was preserved in two cases (with loss of MSH2/MSH6 and isolated MSH6) while 46 cases showed clear nuclear loss of expression; six biopsy cases were inadequate and only one was indeterminate with slightly reduced nuclear expression compared to the internal control. Of the six inadequate cases, one was also inadequate for MLH1 and two were indeterminate for MLH1.

With regard to MSH2 and MSH6, one case showed nuclear expression loss with paired loss of expression of MSH6. One case showed isolated loss of MSH6, while one cased showed loss of MLH1/PMS2 and MSH6 (this was an interesting case which showed true heterogeneity of expression and sub-clonal loss of MSH6 also seen on the resection specimen). No indeterminate or inadequate cases were seen for MSH2 or MSH6. Statistically significant differences were seen between indeterminate/inadequate category for MLH1 versus PMS2 (*p* < 0.0001) and for paired MLH1/PMS2 versus paired MSH2/MSH6 (*p* < 0.0001).

With regard to staining *intensity*, MLH1 and PMS2 (10 and 11 cases, respectively) showed the highest number of weak stains, and this was statistically significant when couples were compared—MLH1/PMS2 versus MSH2/MSH6 (*p* = 0.00198). Score 1 and 2 *patchiness* were seen in only one case each (for MLH1), while no central artefacts were present in any biopsy with any stain.

The 51 cases with loss of MLH1/PMS2 showed *BRAF* V600E mutation in 32 cases, while 17 cases were *BRAF* wild-type; of these latter cases, 13 showed *MLH1* promoter methylation, two were unmethylated, and in two, methylation status was unavailable. In two cases, *BRAF* mutation and *MLH1* methylation status were unavailable. When considering only the 31 cases with indeterminate MLH1 (but loss of PMS2), 19 cases showed *BRAF* V600E mutations while 12 were *BRAF* wild-type cases, nine of which were methylated in *MLH1* promoter, one was indeterminate for methylation (and was lost to follow-up) and two showed no promoter methylation. Of the two *BRAF* wild-type and *MLH1* promoter unmethylated cases, one patient went to genetic testing and no germline mutation was identified, and one patient (a 60-year-old man) refused testing.

### IHC protocol testing in dMMR cases with indeterminate results for MLH1

**Fig. 4:**
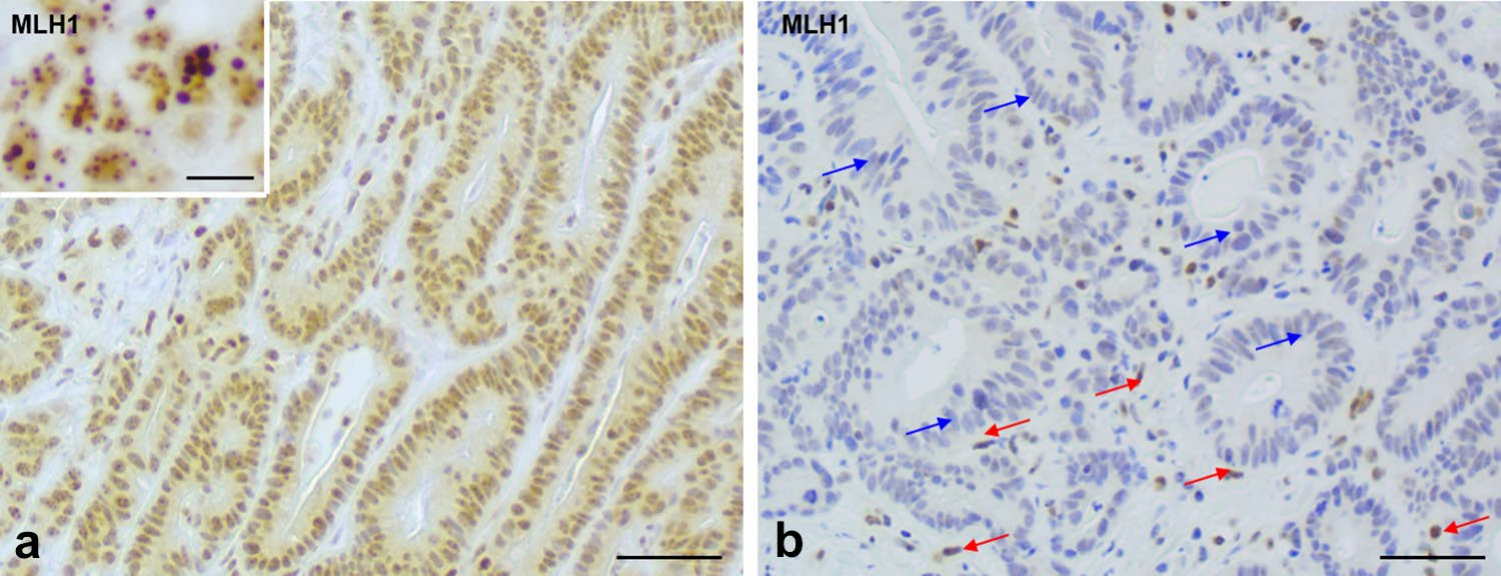
**a** Biopsy case from a MLH1/PMS2 MMR-deficient colorectal cancer showing MLH1 immunostaining with relatively weak nuclear expression and punctate nuclear expression (see inset). **b** This is the same case as **a**, subsequent to modification of immunohistochemistry protocol (protocol A) with reduced primary antibody incubation times. The red arrows show that the internal control cell nuclei have maintained expression while the neoplastic cells show greatly reduced spurious positivity (blue arrows). Scale bar—50 µm, magnification ×40 (10 µm in inset, magnification ×63)

Out of the three modified MLH1 immunostaining protocols, all showed diminished spurious MLH1 staining, with only slight/minimal dot-like nuclear staining, greatly reduced from the initial evaluation with the standard protocol. Comprehensive analysis showed that the best protocol was protocol A with reduced incubation times only. This staining protocol showed the best overall performance, with reduced spurious neoplastic cell MLH1 staining but retained convincing nuclear staining of control, non-neoplastic, nuclei (see Fig. 4). Protocol B still showed neoplastic nuclei MLH1 spurious staining, while protocol C showed excessively diminished control nucleus staining.

### pMMR resection specimens

Forty-eight pMMR biopsies from the prospective series had paired resection specimens, and these all showed preserved nuclear expression; none were classified as indeterminate or inadequate. With regard to staining *intensity*, MLH1 and, more so, PMS2 showed weak nuclear expression, and these differences were statistically significant when comparing all immunostains, both individually and as pairs (*p* = 0.006 and *p* = 0.003, respectively). Significant differences (*p* < 0.0001) were seen in *patchiness* between immunostains, with most score 2 and 3 patchiness seen for MLH1 and PMS2 cases. Similarly, *central artefact* also showed statistically significant differences between immunostains (*p* = 0.0125), with scores 1 and 2 predominantly seen for MLH1 and PMS2.

### dMMR resection specimens

Forty-nine dMMR biopsies (11 from the prospective series and 38 from the retrospective series) had paired resection specimens. MLH1 expression was lost in 44 surgical cases and preserved in three; one case was indeterminate (but showed paired loss of PMS2) and one case was inadequate for both MLH1 and PMS2 (but had had MSI PCR testing on the biopsy showing MSI-H). With regard to staining *intensity* in surgical specimens, both MLH1 and PMS2 showed a significantly higher rate of weak nuclear expression compared to MSH2 and MSH6 (*p* = 0.0006). No statistical differences in *patchiness* were seen between immunostains and, indeed, score 2 or 3 patchiness was seen in 30.9% of all immunoreactions. Of interest, three cases with MLH1/PMS2 loss also showed true heterogeneity, with sub-clonal loss of MSH2 and MSH6 (two cases) or only of MSH6 (one case—already discussed in the dMMR biopsy section). Score 2 *central artefacts* were seen in 26 immunoreactions (13.4%), with no significant differences between antibodies.

### Concordance of MMR status between biopsy and resection specimens

Classification of MMR status, as specified in Table 1, was possible in 92 out of 97 biopsy cases matched with resection specimens, and these were all concordant (47 pMMR and 45 dMMR). In five MMR unclassified cases it was not possible to assign MMR status, as described below. All five cases underwent MSI PCR testing, and four were diagnosed as MSI-H.

In one case, classified as inadequate for MLH1 and PMS2, the matched resection specimen showed loss of MLH1/PMS2 in the tumour. Two biopsy cases were classified as indeterminate for MLH1 and inadequate for PMS2, and these showed loss of MLH1/PMS2 in the matched resection specimen. The last two cases were indeterminate for MLH1 and PMS2, and one was MSI-H at PCR testing and dMMR on the resection specimen, while one was MSS at PCR testing and pMMR on the matched resection specimen.

### Comparison of staining intensity, patchiness and central artefact between matched biopsy and resection specimen samples

No statistically significant differences were seen with regard to staining *intensity* between biopsy and surgical samples when the pair MSH2/MSH6 was considered. On the other hand, intensity was better in biopsy samples stained with MLH1 than on resection specimens, as the former showed more frequent strong staining intensity (*p* < 0.007) while this was not true for PMS2 staining (*p* = 0.9). *Patchiness* was significantly more pronounced in surgical resection specimens than in biopsy samples—score 2/3 patchiness in 6/563 immunoreactions performed on biopsy samples versus 106/380 immunoreactions performed on surgical samples (*p* < 0.0001). *Central artefact* was seen exclusively in resection specimens.

### Other variables

Five rectal adenocarcinomas underwent neoadjuvant treatment (all with pMMR biopsies) and one case had minimal response (score 1 according to Dworak (Dworak et al. 1997), two cases showed moderate score 2 response and two further cases showed near complete score 3 response. No post-surgical pre-treated specimen showed loss of expression (in particular none showed loss of MSH6); one case showed score 3 patchiness.

With regard to histotype evaluated on resection specimens (excluding the five neoadjuvant cases), most MSI cases were solid, rich in infiltrating lymphocytes and mucinous or heterogeneous in histology, while most MSS cases showed conventional, low- or high-grade glandular histology (*p* < 0.0001). Of note is that seven (14%) MSI cases showed conventional low-grade glandular histology. On biopsy specimens, MSI cases were equally distributed between more conventional histology (30/55—54.5%) and mucinous or solid carcinomas (25/55—45.5%), while MSS cases were only rarely solid or mucinous (5/86—5.8%), and these differences were statistically significant (*p* < 0.0001).

Cases deemed adequate for immunoreaction evaluation had a mean of ≥ 5 biopsies, while cases inadequate for immunoreaction evaluation had a mean of ≤ 3 biopsies, although this was not statistically significant (low number of inadequate cases).

## Discussion

Universal MMR/MSI testing in CRC has become widespread in pathology laboratories, as it confers important information regarding prognosis, possible LS diagnosis and treatment choices (Vikas et al. 2023). Recent evidence on neoadjuvant ICI therapy in colonic and rectal cancer requires that testing be performed on biopsy tissue, before surgery. The present study was not focused on whether this was possible—indeed, concordance between MMR status on biopsy and resection specimen is excellent in our study, similarly to prior experiences by other research groups (Kumarasinghe et al. 2010; Shia et al. 2011)—but it concentrated on any MMR technical/evaluation difficulties which may be encountered in the biopsy setting and how to overcome these.

MMR testing on biopsy tissue has important advantages as it enables clinicians to tailor treatment to the patient’s cancer with a rapid (and pre-surgical) evaluation of MMR status. Furthermore, biopsies generally do not suffer from hypo-fixation/cold ischemia and, as shown in our contribution, tend to have little patchiness of expression and no central artefact, both of which are decidedly more pronounced in resection specimens. Biopsy specimens, in our series, showed better quality of expression with fewer weak cases for all stains except for PMS2.

While these results are in some way predictable, the finding of numerous MLH1 indeterminate/problematic cases on biopsy tissue (extremely frequent in our biopsy series—56.4%) has only been briefly touched upon before, and is of extreme importance. Most indeterminate cases showed partial nuclear expression which was either punctate and/or present but of weaker intensity compared to the internal control (so-called MLH1 relatively weak expression cases). This reduced expression was sometimes minimal and could be easily mistaken for preserved MLH1 expression. Punctate expression has been described in a few studies (Niu et al. 2018; Loughrey et al. 2019; Dasgupta et al. 2020; Zhang et al. 2020), and this has been noted to be more obvious in biopsy specimens than in large resection specimens, as underlined by our contribution (Niu et al. 2018; Zhang et al. 2020). Punctate expression of MLH1 has been observed and reported for a specific clone (the M1 clone, Ventana Medical Systems, Roche Diagnostics Division, Hoffmann-La Roche Ltd, Basel, Switzerland), which was also used in our study, and it could represent either increased sensitivity of the clone (which may recognize functionally impaired protein but with preserved antigenicity) or pre-analytic/analytic factors. The UK-NEQAS initiative on quality assurance in immunocytochemistry of MMR protein antibodies, conducted between 2011 and 2019, evaluated more than three different clones for each different antibody. Comprehensive analysis demonstrated that immunoexpression with the DAKO platform and antibodies performed better than Ventana ultraView (see link extension://elhekieabhbkpmcefcoobjddigjcaadp/https://ukneqasiccish.org/wp-content/uploads/2019/09/MMR-Protein-IHC-Parry-and-Dodson-ECP-2019-FINAL.pdf, poster from ECP 2019). Other frequently used clones (such asES05) have not been reported to show punctate MLH1 expression. The study by Zhang et al. (2020) tried to identify the causes of punctate MLH1 expression, postulating that it could be due to short fixation times. However, the pilot study (using colon mucosa samples fixed for various times) failed to demonstrate this. Conversely, in our study, we hypothesized that punctate staining/relatively weak expression MLH1 staining could be due to aggressive antigen retrieval/incubation protocols, and we showed that by decreasing primary antibody incubation times for biopsy samples, this greatly reduces spurious MLH1 expression. This is an important finding as it could mean modification of our IHC protocols, tailoring them to biopsy versus surgical specimens. This finding also underlines the importance of internal quality assurance programmes (Grillo et al. 2017, 2015; Gambella et al. 2017) and that each laboratory must validate and check its immunostaining protocols on different types of tissue.

Reports in the literature have identified punctate/relatively weak expression MLH staining in CRC—as well as in endometrial cancer biopsies (Dasgupta et al. 2020)—and, as testing will be increasingly performed on pre-surgical biopsy samples, it is likely that it will become an important pitfall which must be recognized (Markov et al. 2017).

Of interest is that a relatively recent study (Tarancón-Diez et al. 2020) proposed that sporadic dMMR CRC showed weak to relatively weak MLH1 expression, with complete PMS2 loss in 25%, while this was seen in only 8.5% of LS-associated CRC, making relatively weak expression MLH1 staining a possible marker of sporadic dMMR CRC. In our series, while most cases showed *BRAF* mutation and/or *MLH1* promoter methylation, two biopsy samples were *BRAF* wild-type and *MLH1* promoter unmethylated, and for one patient, LS was excluded by genetic analysis (the other patient refused testing). The idea that relatively weak MLH1 expression could predict CRC sporadic nature is indeed interesting, and our own study seems to lean towards this hypothesis even though further, larger case series are necessary to confirm this finding.

Lastly, an adequate number of neoplastic biopsy samples is the basis of MMR testing, and we have shown that at least five samples is probably sufficient for testing (similarly to the biopsy numbers suggested for HER2 and PD-L1 testing in gastric cancer; Gullo et al. 2015; Grillo et al. 2013; Mastracci et al. 2022). Differences in number of biopsies probably impacts on MMR status definition inasmuch as cases with few biopsies show a lower quantity of invasive cancer available for assessment. Considering that cases are deemed pMMR when > 10% of neoplastic invasive cells show nuclear expression, having a greater number of biopsies, and therefore more neoplastic cells to evaluate, increases confidence in appropriate MMR status assessment.

This study has highlighted four factors which must be considered in this setting: (1) Pathologists must be aware that punctate/dot-like or relatively weak MLH1 expression exists, especially on biopsy tissue if the M1 clone is used. (2) A reliable, strong internal control is important, as cases which are dMMR but show reduced expression compared to the control are sometimes difficult to identify. (3) PMS2 expression is lost in such cases and, while this helps in identifying the cancers as dMMR, it does cause problems for LS diagnosis. Indeed, isolated loss of PMS2, with retained MLH1 expression, triggers referral to genetic analysis due to the increased risk of LS and guides the geneticist to look for PMS2 germline mutations (which is also problematic considering the numerous pseudogenes which increase its complexity). (4) MSI-PCR testing is warranted for biopsies with indeterminate/inadequate MMR results.

In conclusion, evaluation of MMR status on CRC biopsy samples is feasible, and correlation between biopsy and surgical samples is excellent, if pitfalls in interpretation are known. Pathologists must be made aware of punctate/dot-like or relatively weak expression MLH1 immunostaining, which could cause significant problems, and quality assurance programmes to identify appropriate staining protocols, internal to each IHC laboratory, are fundamental for high-quality diagnoses.

## Data Availability

Data will be made available upon reasonable request.
